# Compensatory Base Changes and Varying Phylogenetic Effects on Angiosperm ITS2 Genetic Distances

**DOI:** 10.3390/plants11070929

**Published:** 2022-03-30

**Authors:** Ruixin Cao, Shuyan Tong, Tianjing Luan, Hanyun Zheng, Wei Zhang

**Affiliations:** Marine College, Shandong University, 180 Wenhua Xilu, Weihai 264209, China; ruixincao611@gmail.com (R.C.); t243382368@163.com (S.T.); 201800810150@mail.sdu.edu.cn (T.L.); hyun200004@163.com (H.Z.)

**Keywords:** compensatory base change, genetic distance, ITS2, RNA substitution model, secondary structure, sister species pairs

## Abstract

A compensatory base change (CBC) that coevolves in the secondary structure of ribosomal internal transcribed spacer 2 (ITS2) influences the estimation of genetic distance and thus challenges the phylogenetic use of this most popular genetic marker. To date, however, the CBC effect on ITS2 genetic distance is still unclear. Here, ITS2 sequences of 46 more recent angiosperm lineages were screened from 5677 genera and phylogenetically analyzed in sequence-structure format, including secondary structure prediction, structure-based alignment and sequence partition of paired and unpaired regions. ITS2 genetic distances were estimated comparatively by using both conventional DNA substitution models and RNA-specific models, which were performed in the PHASE package. Our results showed that the existence of the CBC substitution inflated the ITS2 genetic distances to different extents, and the deviation could be 180% higher if the relative ratio of substitution rate in ITS2 secondary structure stems was threefold higher than that in the loops. However, the CBC effect was minor if that ratio was below two, indicating that the DNA model is still applicable in recent lineages in which few CBCs occur. We thus provide a general empirical threshold to take account of CBC before ITS2 phylogenetic analyses.

## 1. Introduction

Genetic distance is conventionally represented by the number of nucleotide differences between two sequences that derive from a common ancestor [1]. It is an essential parameter in the study of molecular evolution for calculating the evolutionary rate, estimating divergence, and inferring phylogeny among genes or organisms [2]. Therefore, it is vital to acquire reliable genetic distances.

An important issue related to the calculation of genetic distances is the estimation of the pattern of nucleotide substitution. The basic principle of phylogenetic inference assumes that two sequences derived from a common ancestor will substitute independently and randomly and eventually diverge from each other [1,3]. It is acknowledged that when a sequence evolves rapidly, multiple substitutes are likely to occur and erase any prior substitution record. As evolutionary time elapses, multiple substitutes will accumulate, and the observed distance will become increasingly deviated and eventually saturated, with the result that late substitutions have little or no impact on the total number of the observed nucleotide differences [3,4,5]. In order to adjust the site-change underestimation caused by the multiple substitutes, several models of DNA substitution have been proposed [6].

**Figure 1:**
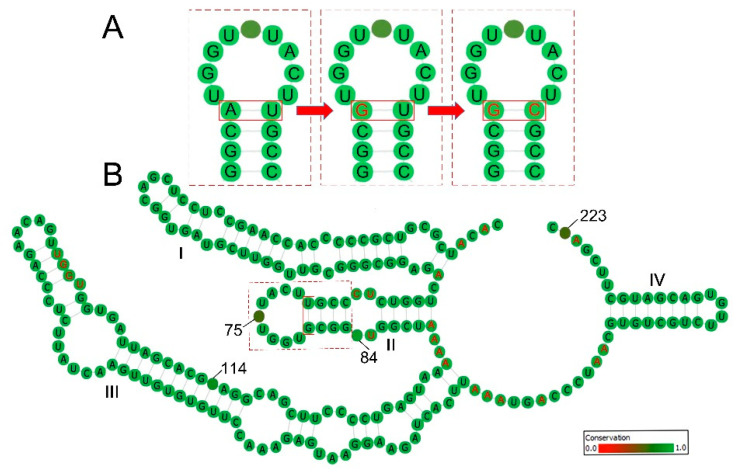
ITS2 secondary structures and the compensatory base change (CBC) on their base pairs. (**A**) A snapshot of ITS2 stem II showing how CBC occurs on RNA secondary structure; (**B**) an example of ITS2 consensus secondary structure derived from the sister species pair of *Sinosenecio bodinieri* and *S. confervifer*. The four stems are labelled I–IV. The pyrimidine–pyrimidine bulge in stem II, the UGGU in stem III and the high adenine content between stems that are typical of nearly all angiosperm ITS2 secondary structures are indicated in red. The degree of conservation over the entire alignment is displayed in color grades from green (conservative) to red (variable), and the variable bases are labeled with site numbers.

In contrast to the site-change underestimation, it is worth noting that some site changes can also be amplified through covariation. One of the covariation patterns is known as the compensatory base change (CBC), and this often occurs in structural RNA regions, wherein substitutions on one side of a pair are compensated by substitutions on the other side in order to restore the RNA structure stability and function (Figure 1A) [7,8,9].

Like multiple substitutions, CBCs also violate the basic assumption of independent and random substitution. Some authors are concerned that neglecting the CBC effect could result in counting the same substitution twice and could thus overestimate genetic distances and mislead phylogenetic inference [10,11,12]. Accordingly, some RNA-specific substitution models have been proposed to account for the covariable base states together in both sides instead of treating them separately in each side [13,14,15]. Through their practical applications, some authors found that the phylogeny of ancient lineages constructed using the RNA model outperforms that of the DNA model due to its shorter branch length and higher likelihoods [16,17]. In contrast, other studies in recent lineages showed a small empirical effect of CBC in phylogenetic analyses [9]. Moreover, other studies indicated that using the RNA model could reduce the weights of stem substitutions, which is consequently equivalent to up-weighting loop substitutions. However, the loop regions are more liable to be saturated and misaligned and thus more likely to lead to an inaccurate phylogeny [18]. Taken together, although the CBC effect has been acknowledged, the extent of its effect on genetic distance in different lineages still needs critical assessment. 

The ribosomal internal transcribed spacer 2 (ITS2) is an ideal region to assess CBC effects on genetic distance. First, ITS2 has been widely used in plant phylogeny and DNA barcoding and has accumulated millions of sequences in GenBank, and thereby could facilitate large dataset analyses across a wide range of taxa. Second, despite rapidly evolving, ITS2 has a highly conserved secondary structure throughout Eukaryota, indicating that this region is under functional constraints. Accumulating evidence in the study of ribosome biogenesis shows that ITS2 is the last spacer removed from the 5.8S-ITS2-25S complex (27SB pre-rRNA in yeast), in which ITS2 folding could bring 5.8S and 25S pre-rRNA together to form a hallmark ‘foot’ architecture during ribosome assembly. The characteristic ITS2 secondary structure, which is always in “hairpin” or “ring-pin” form, functions as a scaffold to mediate this ‘foot’ topological rearrangement and is thus necessary for the downstream ribosome assembly [19,20,21]. The availability of this ITS2 secondary structure will contribute significantly to accounting for the true number of ITS2 substitutions using structure-based RNA models [9,21]. In this study, we focused on the phylogenetic implications of CBC substitution and quantified its effects on ITS2 genetic distance by comparatively using both DNA- and RNA-specific models. Furthermore, it has been considered that evolutionary saturation and misalignment are likely to occur in ancient lineages, which may mislead substitution counting. We thus sampled lineages including closely related species using the large dataset of ITS2. This also allowed us to explore the minimum genetic distance between species and test the hypothesis of the ITS2 molecular threshold [22].

## 2. Results

### 2.1. ITS2 Sequences and Their Genetic Distances among the Investigated Lineages

For the investigated lineages, all sequences annotated “internal transcribed spacer 2” or “internal transcribed spacer” that had been deposited in GenBank before April 2019 were retrieved for analysis. We excluded incomplete ITS2 sequences and lineages with an insufficient number of species. A total of 99,128 ITS2 sequences representing 5677 genera and 16,371 species were initially obtained. These sequences of a genus were each constructed into a neighbor-joining (NJ) tree. We then deleted genera with poor species resolution or lineages with paraphyletic or polyphyletic species. Finally, a total of 46 sister species pairs (SSPs) were identified, involving 20 genera and 16 families (Table S1). 

Distphase analysis in the PHASE package showed that the ITS2 genetic distances based on DNA models (GD_DNA_) of these 46 SSPs ranged widely, from 0.12% to 8.72%, with an average value of 2.54%. Distance analysis using MEGA software yielded an almost identical value as PHASE for each SSP (paired-samples *t* test, *p* = 0.443), and accordingly, the minimum, maximum and average values were 0.12%, 8.78% and 2.53%, respectively (Table S1). These results validated the GD_DNA_, which was used for comparison with GD_RNA_ in the following analyses. Although the GD_DNA_ varied greatly in a few extreme cases, 80% of the lineages’ GD_DNA_ was less than 4.0% (Figure S1).

### 2.2. ITS2 Secondary Structure and the Structure-Based SSP Alignments

The consensus ITS2 secondary structure of each SSP had a typical “four-helix model” (Figure 1B), of which helix III was the longest and had the UGGU motif, the helix II was rich in G-C pairs and had a pronounced pyrimidine bulge, helixes I and IV were relatively variable in their length and base pair composition. The loops between the four stems had a characteristic adenine bias. These common features validate the ITS2 secondary structure prediction in this study.

Based on these consensus secondary structures, the ITS2 sequences were partitioned into paired and unpaired regions. The aligned length of ITS2 alignments ranged from 165 to 264 bp, with an average length of 231 bp, including 134 bp paired and 97 bp unpaired regions in the SSP consensus secondary structures (Table S1). On average, nearly 60% of ITS2 bp were involved in the stems, in which CBC substitution occurs, indicating that RNA models may be more appropriate for ITS2 than the conventional DNA models. Taken across all 46 SSP alignments, a total of 332 variable sites were observed, including 77 stem sites and 255 loop sites. The variable sites in the stems of each SSP consensus secondary structure ranged from seven to zero, and they were generally less than their loop sites (Table S1). We found that 13 of 46 SSP alignments had no variable sites in the stem region (Table S1) and removed them to make the RNA model applicable. In addition, there were three SSP alignments that had no variable sites in their loop regions, and the RNA models were also not applicable to these. When all of these inapplicable SSP alignments were removed, the remaining 30 were used for the PHASE RNA model.

### 2.3. Comparison of the Best-Fitting DNA and RNA Models

**Figure 2:**
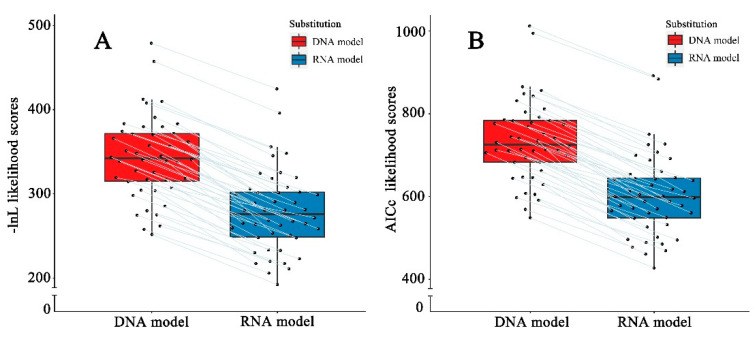
Comparisons of distinct likelihoods obtained from the best-fitting DNA and RNA models. (**A**) -ln L likelihood; (**B**) AICc likelihood. The same ITS2 sequence-structure alignments analyzed separately with DNA and RNA-models are connected with lines.

The structural partitioning of ITS2 alignments allows loops and stems to be tested separately via distinct substitution model parameters in the RNA models, which assigned a DNA model to loop sites and an RNA-specific model to stem sites (Table S3). The Perl script implemented in the likelihood program of PHASE was executed to adjust these model parameters to make distinct model test results compatible. These model test results showed that for each ITS2 alignment the best-fit RNA model was always lower than the best-fit DNA model in either log-likelihood (-ln L) or AICc scores (Figure 2), averaging 81% and 82% of the DNA model, respectively (Table S3). Notably, the best-fit RNA models in most lineages were consistent with each other, among which 85% (39/46 lineages) of the ITS2 stems evolved homogeneously under the RNA7G model (Table S3). Given that both -ln L and AICc values are associated with branch lengths on a given tree, and branch length can be represented as genetic distance, it is reasonable to speculate that GD_RNA_ should be lower than GD_DNA_ in these lineages.

### 2.4. Comparison between GD_RNA_ and GD_DNA_

Phylogenetic analyses of ITS2 sequence-structure alignments using the best-fit DNA model (for unpaired regions) and RNA model (for paired regions) generated the GD_RNA_. The PHASE analysis showed that the ITS2 GD_RNA_ of these 30 SSP alignments ranged from 0.12% to 25.28%. In contrast, the ITS2 GD_DNA_ ranged from 0.53% to 8.29%. Unexpectedly, the average value of GD_RNA_ was higher than that of GD_DNA_ for 26/30 SSP alignments, averaging 174% of the GD_DNA_ (5.76% ± 5.50% vs. 3.04% ± 2.02%; Table S2), indicating that some constraints have limited PHASE analysis when using RNA models.

**Figure 3:**
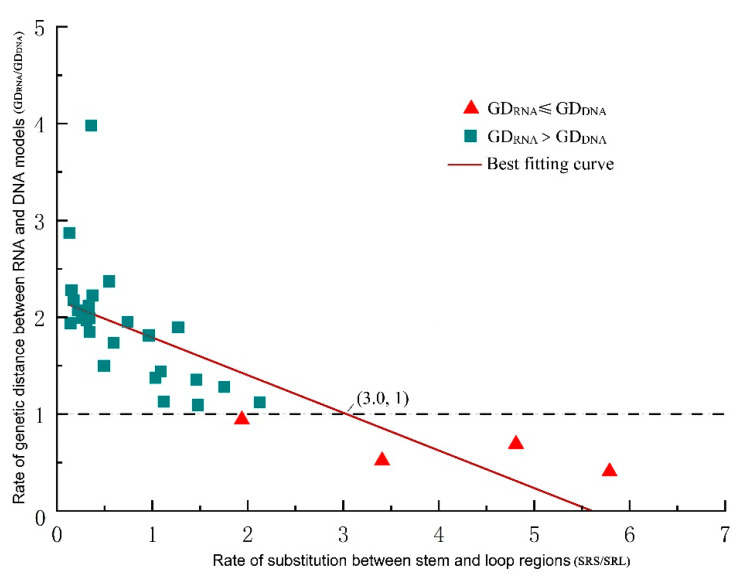
A scatter plot showing the effect of compensatory base change on genetic distance. As the rate of substitution between stem and loop regions increases, the rate of genetic distance between RNA and DNA models becomes less and less, making RNA models more and more effective and play a leading role when the substitution rate ratio is greater than three.

Given that the genetic distance depends on the substitutions within base pairs, we investigated the substitution sites in each ITS2 partition (loop vs. stem regions) separately, counting and comparing their respective raw substitution rates. We found that the GD ratio (GD_RNA_*/*GD_DNA_) decreased as the SRS/SRL ratio (substitution rate in stems/substitution rate in loops) increased. When the SRS/SRL ratio equaled 2.0, the expected result, that GD_RNA_ was lower than GD_DNA_, began to appear. When the SRS/SSL ratio rose to 3.0, GD_RNA_ was always smaller than GD_DNA_ (Figure 3).

## 3. Discussion

An accurate estimation of genetic distance is a prerequisite for molecular phylogeny, molecular chronogram and evolution, which are all based on the measurement of sequence divergences [1]. However, genetic distance estimation is not easy for the ITS2 region, although it has been widely used as a phylogenetic marker [21,23,24]. In vivo, ITS2 rRNA folds and functions in the form of a secondary structure that is maintained through base-pair interactions [19,20]. Our results showed that ITS2 secondary structures are consistent with the typical “four-helix” model across a broad range of 13 orders, confirming that ITS2 is under evolutionary constraints through CBC substitution, to maintain the specific secondary structures that provide functionality [21]. We found that nearly 60% of ITS2 bp were involved in the stem, where the CBC substitution occurs. In addition, model testing showed that, for all 46 SSP alignments, the RNA substitution models always had a higher likelihood than the conventional DNA models (Table S3). Taken together, our results corroborate the expectation that base-pair covariation has occurred in ITS2 within the study lineage [25]. Therefore, the RNA model should be considered in genetic distance calculations to account for this non-independent CBC substitution [13,14,15,17].

The distinct evolutionary pattern between stem and loop regions should be considered seriously in genetic distance analyses. Some authors have warned that the loops are more apt to evolutionary saturation and/or misalignment in ancient lineages, wherein using RNA models down-weights stems and virtually magnifies the loop effect and thus misleads phylogenetic signals [18]. This view has been confirmed here by our finding that ITS2 loop sites are more variable than stem sites (Table S2). To avoid the possible loop effect on stem phylogenetic analyses, this study chose the most recent lineage and focused on sequence divergence between sister species. Thereby, we could also explore the ITS2 threshold among species based on the RNA divergence. We found that although the GD_DNA_ varied greatly in a few extreme cases, 80% of lineages’ GD_DNA_ was less than 4.0%, consistent with the previous study of Qin et al. [22]. In general, the almost identical results between PHASE and the conventionally used MEGA validate the GD_DNA_ of SSP alignments and the usability of PHASE. Taken across all six lineages within the best scope of application through PHASE (Figure S2), all the GD_RNA_ were lower than GD_DNA_, averaging 56% of the GD_DNA_ (1.18% vs. 2.11%). This result justifies some authors’ concern that failing to account for the covariation pattern (CBC) of stem regions could result in an overestimation of phylogenetic variation and leads to misleading distance-based statistics with strong support [12,13]. Furthermore, this study provided an empirical estimation that was 180% higher when this non-independence was neglected.

A highlight of this study is providing an empirical threshold of a threefold substitution rate between the stem region and loop region to help determine when it is necessary to take account of the CBC effect. Because, although CBC substitutions affect the DNA-based phylogenetic analyses, not all the substitutions in the stem region are of the CBC pattern. In fact, CBC has generally been considered a two-step substitution through a slightly unstable intermediate base-pair; for example, the substitution from AU to GC is mainly through a GU intermediate, as shown in stem II of Figure 1 [7,9,25]. It has been revealed that the time required for these changes spans one or several closed related species on a phylogenetic tree [8,9]. Within these time constraints, despite variations occurring in the stem region, they are only one-side substitutions before the CBCs and thus still obey the site-independence assumption. In this context, calculating genetic distances using DNA models seems unlikely to be problematic within such recently diverged lineages, in which few CBCs are observed [9]. However, as lineages diverge further, the CBC substitutions become common, and the DNA models are less able to describe these variations. Therefore, the threshold we provide here could contribute to clarifying how much of the variation present in stem regions could affect the estimation of genetic distance.

## 4. Materials and Methods

### 4.1. Lineage Sampling and Sister Species Pair Acquisition

The species validity and coverage within a certain lineage (genus) were based on Plant List (online service http://www.theplantlist.org, accessed on 20 January 2021). We sampled lineages from GenBank for which the complete ITS2 sequences were available from at least one half of the total species, and at least two sequences available per species. The ITS2 region was identified and delimited from the raw sequences using GenBank annotations and the “ITS2 annotation” online service in the ITS2 database (http://its2.bioapps.biozentrum.uni-wuerzburg.de, accessed on 15 February 2021). The ITS2 matrix was aligned with MAFFT, using the G-INS-i iterative refinement method and the default parameters (Scoring matrix: 200PAM/k = 2; Gap opening penalty: 1.53; Offset value: 0) [26]. Then, the aligned sequence matrix was imported into MEGA11 [27] to construct the neighbor joining (NJ) tree based on the Kimura 2-parameter (K2P) model, with the following options: substitutions included transitions and transversions, uniform rates and homogeneous pattern, and gaps/missing data were treated as complete deletion. The confidence of the tree branch was evaluated using 1000 replicates. The lineages were screened again based on the tree topology, which met the following criteria. First, species resolution was at least 50% on the ITS2 NJ tree. Second, species with multiple individuals clustering together into a monophyletic group in NJ trees with a bootstrap value above 50% were regarded as successful species identifications. Third, the shallow phylogenies (i.e., clades toward the tips) were well-resolved, and at least one sister species pair (SSP) was identified.

### 4.2. ITS2 Sequence-Structure Alignment 

The individual ITS2 sequence of SSPs was folded into a secondary structure using homology modeling from the online ITS2 database [28] and was exported into the Vienna format. Then, a raw sequence-structure matrix which was composed of every single ITS2 sequence, and its secondary structure was synchronously aligned using 4SALE 1.7 [29,30]. After manual adjustment using the 4SALE editor, the ITS2 consensus secondary structure was yielded into a graphical form. The 75% majority consensus secondary structure was selected and transformed manually into the Vienna format for subsequent analyses. By referring to the consensus secondary structure, the ITS2 sequence matrix was partitioned into paired and unpaired regions and was phylogenetically analyzed both separately and in combination using RNA and DNA models.

### 4.3. Genetic Distance Acquisition Using DNA and RNA Substitution Models

The best-fitting model for the ITS2 sequence-structure (RNA form) evolution was estimated using the optimizer in PHASE package 3.0 [15,31], wherein a total of 2 × 16 mixed models were tested (REV or HKY85 for loop regions and 16 base-paired models for stem regions) [15]. Considering that different numbers of parameters between the 4-, 7-, and 16-state treatments of base state in the mixed models could mislead the comparison of different likelihood values, we used a Perl script for likelihood correction to make model test results compatible [15]. The REV or HKY85, the best fitting in the mixed model test, was also used for calculating genetic distances of ITS2 sequence. Then, genetic distance based on RNA and DNA models was calculated separately through distphase in the PHASE package. Statistical analyses were then performed to summarize these results using Microsoft 2016 Excel, SPSS 22.0 and Origin Pro 9.0.

## Data Availability

Not applicable.

## Supplementary Materials

The following supporting information can be downloaded at: https://www.mdpi.com/article/10.3390/plants11070929/s1, Figure S1: ITS2 genetic distances of sister species pairs vary among different lineages; Figure S2: Comparisons of ITS2 genetic distances between RNA and DNA substitution models when the effect of compensatory base change in stem regions is too large to be neglected; Table S1: Statistics of phylogenetic analyses from all sequence-structure matrices; Table S2: Statistics of phylogenetic analyses from the 30 sequence-structure matrices that can be applicable for the RNA substitution models. Table S3: Comparison of likelihood scores between DNA- and RNA-specific models applied to the ITS2 alignments.
