# Supplementary material for: Compensatory Base Changes and Varying Phylogenetic Effects on Angiosperm ITS2 Genetic Distances
Source: Plants (Basel). 2022 Mar 30;11(7):929. doi: 10.3390/plants11070929 (PMC9003356; doi:10.3390/plants11070929)
Supplement: Supplementary file 1 [file plants-11-00929-s001.zip › plants-1613253-supplementary.pdf]

Supplementary Materials

1 Supplementary Tables

Table S1. Statistics of phylogenetic analyses from all sequence-structure matrices[a]

Family	Genus	SSP	GD_{RNA} (PHASE)	GD_{DNA} (PHASE)	GD_{DNA} (MEGA)	GD_{RNA}/ GD_{DNA}	Matrix length (bp)	Unpaired bases (bp)	Base pairs (bp)	No. of VSL	No. of VSS
**Araceae**	*Acorus*	*americanus-calamus*	0.0486	0.0235	0.0217	2.0713	201	71	65	5	1
**Asteraceae**	*Adenocaulon*	*chilense-lyratum*	0.0732	0.0404	0.0413	1.8134	227	101	63	5	3
		bicolor-nepalense	0.0132	0.0191	0.0213	0.6909	227	101	63	1	3
	Hirpicium	*gazanioides-gorterioides*	0.0950	0.0490	0.0445	1.9396	221	97	62	11	1
		linearifolium-armerioides	0.0527	0.0303	0.0307	1.7380	220	82	69	6	3
	Sinosenecio	*chienii-sichuanicus*	0.1711	0.0596	0.0567	2.8716	225	107	59	14	1
		bodinieri-confervifer	0.0193	0.0099	0.0099	1.9559	224	92	66	3	1
		guangxiensis_saxatilis	0.0546	0.0274	0.0277	1.9917	225	99	63	6	1
		homogyniphyllus-yilingii	0.0981	0.0502	0.0488	1.9530	225	103	61	16	7
		leiboensis-denticulatus	0.0174	0.0159	0.0159	1.0947	226	96	65	2	2
		newcombei-koreanus	0.0335	0.0120	0.0122	2.7838	225	83	71	3	0

**Brassicaceae**	*Moricandia*	*arvensis-spinosa*	0.0000	0.0026	0.0027	0.0038	192	86	53	0	1
**Celastraceae**	*Celastrus*	*monospermoides-hindsii*	0.0307	0.0116	0.0105	2.6515	221	95	63	6	0
		monospermus-virens	0.0920	0.0404	0.0412	2.2807	236	102	67	10	1
		paniculatus-pringlei	0.0095	0.0101	0.0101	0.9425	221	93	64	3	4
**Ceratophyllaceae**	*Ceratophyllum*	*demersum-platyacanthum*	0.0162	0.0081	0.0082	1.9975	260	132	64	8	0
		submersum-muricatum	0.0290	0.0137	0.0134	2.1162	256	116	70	5	1
**Euphorbiaceae**	*Plukenetia*	*huayllabambana-volubilis*	0.0121	0.0107	0.0105	1.1283	233	123	55	2	1
**Fabaceae**	*Delonix*	*floribunda-pumila*	0.0229	0.0096	0.0097	2.3709	201	71	65	2	1
	Caragana	*bongardiana-tragacanthoides*	0.0000	0.0023	0.0023	0.0043	219	77	71	0	1
		opulens-licentiana	0.0000	0.0012	0.0012	0.0086	218	74	72	0	1
		spinosa-erinacea	0.0574	0.0187	0.0189	3.0619	219	87	66	4	0
**Gesneriaceae**	*Primulina*	*pungentisepala-ningmingensis*	0.0100	0.0053	0.0053	1.8982	242	94	74	1	1
		chizhouensis-linglingensis	0.0154	0.0137	0.0138	1.1236	246	102	72	2	3
		huangii-repanda	0.0648	0.0291	0.0292	2.2260	246	88	79	6	2
		langshanica-xinningensis	0.0964	0.0443	0.0447	2.1754	251	117	67	10	1
		tenuituba-vestita	0.0207	0.0153	0.0154	1.3570	256	108	74	3	3

		alutacea-suichuanensis	0.0119	0.0082	0.0082	1.4416	247	87	80	1	1
	Nematanthus	*bradei-serpens*	0.0113	0.0045	0.0045	2.5112	224	90	67	1	0
**Melastomataceae**	*Antherotoma*	*naudinii-phaeotricha*	0.0085	0.0030	0.0030	2.8802	227	79	74	1	0
**Moraceae**	*Maclura*	*amboinensis-fruticosa*	0.0640	0.0260	0.0229	2.4583	241	107	67	7	0
		brasiliensis-pomifera	0.1061	0.0420	0.0425	2.5258	237	99	69	11	0
**Palmae**	*Wallichia*	*marianneae-gracilis*	0.0251	0.0077	0.0077	3.2837	264	82	91	3	0
**Papaveraceae**	*Meconopsis*	*betonicifolia-grandis*	0.0399	0.0178	0.0178	2.2447	251	115	68	7	0
		concinna-delavayi	0.0913	0.0458	0.0461	1.9914	256	118	69	10	2
		georgei_pseudovenusta	0.0449	0.0243	0.0243	1.8476	252	116	68	5	1
		lancifolia-impedita	0.0417	0.0303	0.0304	1.3748	252	128	62	8	4
		primulina-wumungensis	1.5630	0.0872	0.0878	17.9347	252	138	57	20	0
		quintuplinervia-punicea	0.0317	0.0161	0.0162	1.9682	252	110	71	5	1
		torquata-discigera	0.1244	0.0829	0.0842	1.5000	253	137	58	24	5
**Ranunculaceae**	*Dichocarpum*	*basilare-trifoliolatum*	0.0146	0.0048	0.0048	3.0438	209	69	70	1	0
**Santalaceae**	*Buckleya*	*graebneriana-distichophylla*	0.2528	0.0635	0.0643	3.9822	165	69	48	8	2
		henryi-lanceolata	0.0874	0.0681	0.0676	1.2838	173	73	50	5	6
**Sapotaceae**	*Planchonella*	*howeana-myrsinodendron*	0.0148	0.0364	0.0364	0.4065	229	67	81	1	7

		torricellensis-polyneura	0.0098	0.0189	0.0191	0.5192	231	69	81	1	4
**Solanaceae**	*Markea*	*huilensis-lopezii*	0.0203	0.0089	0.0090	2.2843	227	109	59	2	0
**Average**			0.0808	0.0254	0.0253	2.2116	231	97	67	6	2

[a] SSP is the abbreviation of sister-species pair; GD_{DNA} and GD_{RNA} represent the genetic distance generated by DNA and RNA substitution models, respectively. VSL and VSS represent variable sites in loops and stems, respectively.

Table S2. Statistics of phylogenetic analyses from the 30 sequence-structure matrices that can applicable for the RNA substitution models[a]

Family	Genus	SSP	GD_{RNA}	GD_{DNA}	GD_{RNA}/ GD_{DNA}	Unpaired bases (bp)	No. of VSL	SRL	Base pairs (bp)	No. of VSS	SRS	SRS/ SRL
**Santalaceae**	*Buckleya*	*graebneriana-distichophylla*	0.2528	0.0635	3.9822	69	8	0.1159	48	2	0.0417	0.3594
**Asteraceae**	*Sinosenecio*	*chienii-sichuanicus*	0.1711	0.0596	2.8716	107	14	0.1308	59	1	0.0169	0.1295
**Fabaceae**	*Delonix*	*floribunda-pumila*	0.0229	0.0096	2.3709	71	2	0.0282	65	1	0.0154	0.5462
**Celastraceae**	*Celastrus*	*monospermus-virens*	0.0920	0.0404	2.2807	102	10	0.098	67	1	0.0149	0.1522
**Gesneriaceae**	*Primulina*	*huangii-repanda*	0.0648	0.0291	2.226	88	6	0.0682	79	2	0.0253	0.3713
**Gesneriaceae**	*Primulina*	*langshanica-xinningensis*	0.0964	0.0443	2.1754	117	10	0.0855	67	1	0.0149	0.1746
**Ceratophyllaceae**	*Ceratophyllum*	*submersum-muricatum*	0.0290	0.0137	2.1162	116	5	0.0431	70	1	0.0143	0.3314
**Araceae**	*Acorus*	*americanus-calamus*	0.0486	0.0235	2.0713	71	5	0.0704	65	1	0.0154	0.2185
**Asteraceae**	*Sinosenecio*	*guangxiensis_saxatilis*	0.0546	0.0274	1.9917	99	6	0.0606	63	1	0.0159	0.2619
**Papaveraceae**	*Meconopsis*	*concinna-delavayi*	0.0913	0.0458	1.9914	118	10	0.0847	69	2	0.029	0.342
**Papaveraceae**	*Meconopsis*	*quintuplinervia-punicea*	0.0317	0.0161	1.9682	110	5	0.0455	71	1	0.0141	0.3099
**Asteraceae**	*Sinosenecio*	*bodinieri-confervifer*	0.0193	0.0099	1.9559	92	3	0.0326	66	1	0.0152	0.4646
**Asteraceae**	*Sinosenecio*	*homogyniphyllus-yilingii*	0.0981	0.0502	1.953	103	16	0.1553	61	7	0.1148	0.7387
**Asteraceae**	*Hirpicium*	*gazanioides-gorterioides*	0.0950	0.049	1.9396	97	11	0.1134	62	1	0.0161	0.1422

**Gesneriaceae**	*Primulina*	*pungentisepala-ningmingensis*	0.0100	0.0053	1.8982	94	1	0.0106	74	1	0.0135	1.2703
**Papaveraceae**	*Meconopsis*	*georgei_pseudovenusta*	0.0449	0.0243	1.8476	116	5	0.0431	68	1	0.0147	0.3412
**Asteraceae**	*Adenocaulon*	*chilense-lyratum*	0.0732	0.0404	1.8134	101	5	0.0495	63	3	0.0476	0.9619
**Asteraceae**	*Hirpicium*	*linearifolium-armerioides*	0.0527	0.0303	1.738	82	6	0.0732	69	3	0.0435	0.5942
**Papaveraceae**	*Meconopsis*	*torquata-discigera*	0.1244	0.0829	1.5	137	24	0.1752	58	5	0.0862	0.4921
**Gesneriaceae**	*Primulina*	*alutacea-suichuanensis*	0.0119	0.0082	1.4416	87	1	0.0115	80	1	0.0125	1.0875
**Papaveraceae**	*Meconopsis*	*lancifolia-impedita*	0.0417	0.0303	1.3748	128	8	0.0625	62	4	0.0645	1.0323
**Gesneriaceae**	*Primulina*	*tenuituba-vestita*	0.0207	0.0153	1.357	108	3	0.0278	74	3	0.0405	1.4595
**Santalaceae**	*Buckleya*	*henryi-lanceolata*	0.0874	0.0681	1.2838	73	5	0.0685	50	6	0.12	1.752
**Euphorbiaceae**	*Plukenetia*	*huayllabambana-volubilis*	0.0121	0.0107	1.1283	123	2	0.0163	55	1	0.0182	1.1182
**Gesneriaceae**	*Primulina*	*chizhouensis-linglingensis*	0.0154	0.0137	1.1236	102	2	0.0196	72	3	0.0417	2.125
**Asteraceae**	*Sinosenecio*	*leiboensis-denticulatus*	0.0174	0.0159	1.0947	96	2	0.0208	65	2	0.0308	1.4769
**Celastraceae**	*Celastrus*	*paniculatus-pringlei*	0.0095	0.0101	0.9425	93	3	0.0323	64	4	0.0625	1.9375
**Asteraceae**	*Adenocaulon*	*bicolor-nepalense*	0.0132	0.0191	0.6909	101	1	99	63	3	0.0476	4.8095
**Sapotaceae**	*Planchonella*	*torricellensis-polyneura*	0.0098	0.0189	0.5192	69	1	0.0145	81	4	0.0494	3.4074
**Sapotaceae**	*Planchonella*	*howeana-myrsinodendron*	0.0148	0.0364	0.4065	67	1	0.0149	81	7	0.0864	5.7901

Average		0.0576± 0.0550	0.0304± 0.0202	1.7351± 0.7153	98	6

[a] The table is listed by the proportion between GD_{RNA} and GD_{DNA} in descending order. SSP is the abbreviation of sister-species pair; GD_{DNA} and GD_{RNA} represent the genetic distance generated by DNA and RNA substitution models, respectively. VSL and VSS represent variable sites in loops and stems, respectively. SRL and SRS represent substitution rate in loops and stems, respectively.

Table S3. Comparison of likelihood scores between DNA- and RNA-specific models applied to the ITS2 alignments

Genus	SSP	DNA model			Mixed model (DNA model_ RNA model)		
		Model name	-lnL	AICc	Model name	-lnL	AICc
Acorus	americanus-calamus	HKY85+G_HKY85+G	274.65	711.9955	HKY85+G_RNA7G.equal	219.89	600.4076
Wallichia	marianneae-gracilis	HKY85+G_HKY85+G	351.22	735.2527	HKY85+G_RNA7G.equal	267.50	565.7082
Antherotoma	naudinii-phaeotricha	HKY85+G_HKY85+G	285.36	607.6319	HKY85+G_RNA7G.equal	217.12	469.0643
Buckleya	graebneriana-distichophylla	HKY85+G_HKY85+G	261.83	568.9204	HKY85+G_RNA7G.equal	217.27	477.6981
	henryi-lanceolata	HKY85+G_HKY85+G	279.94	605.0870	HKY85+G_RNA7G.equal	222.92	488.9529
Celastrus	monospermoides-hindsii	REV+G_HKY85+G	318.53	710.5195	HKY85+G_RNA7G.equal	262.70	588.4770
	monospermus-virens	HKY85+G_HKY85+G	348.06	741.0162	HKY85+G_RNA7G.equal	281.28	605.3768
	paniculatus-pringlei	HKY85+G_HKY85+G	316.63	694.3475	HKY85+G_RNA7G.equal	259.74	578.5131

**Ceratophyllum**	demersum-platyacanthum	REV+G_HKY85+G	390.77	842.6233	HKY85+G_RNA7E.equal	325.27	707.4912
	submersum-muricatum	HKY85+G_HKY85+G	383.25	831.4617	HKY85+G_RNA7G.equal	318.32	699.5474
**Adenocaulon**	chilense-lyratum	HKY85+G_HKY85+G	337.66	712.2669	HKY85+G_RNA7G.equal	280.76	596.3509
	bicolor-nepalense	HKY85+G_HKY85+G	356.42	777.9506	HKY85+G_RNA7G.equal	298.86	660.7603
**Hirpicium**	gazanioides-gorterioides	HKY85+G_HKY85+G	361.73	768.3883	HKY85+G_RNA7G.equal	301.28	645.4012
	linearifolium-armerioides	HKY85+G_HKY85+G	345.09	731.1369	HKY85+G_RNA7G.equal	280.65	600.1597
**Sinosenecio**	chienii-sichuanicus	HKY85+G_HKY85+G	379.69	804.2640	HKY85+G_RNA7G.equal	324.51	691.8382
	bodinieri-confervifer	REV+G_HKY85+G	325.49	720.3781	HKY85+G_RNA7G.equal	264.58	588.1793
	guangxiensis_saxatilis	HKY85+G_HKY85+G	343.59	744.2114	HKY85+G_RNA16C	280.21	627.8520
	homogyniphyllus-yilingii	HKY85+G_REV+G	457.29	1012.2133	HKY85+G_RNA16C	396.02	891.7410
	leiboensis-denticulatus	HKY85+G_HKY85+G	327.39	699.7014	HKY85+G_RNA7G.equal	263.85	570.5449
	newcombei-koreanus	HKY85+G_HKY85+G	317.00	682.9936	HKY85+G_RNA7E.equal	248.06	549.2989
**Planchonella**	howeana-myrsinodendron	HKY85+G_HKY85+G	340.20	713.3442	HKY85+G_RNA16C	258.53	560.6818

	torricellensis-polyneura	HKY85+G	340.67	705.7608	HKY85+G_RNA7E.equal	253.34	547.8286
**Primulina**	pungentisepala-ningmingensis	HKY85+G_HKY85+G	338.61	714.0628	HKY85+G_RNA7G.equal	268.71	572.1788
	chizhouensis-linglingensis	HKY85+G_HKY85+G	357.16	751.1173	HKY85+G_RNA7G.equal	288.77	612.2504
	huangii-repanda	HKY85+G_HKY85+G	370.37	773.5772	HKY85+G_RNA7G.equal	290.62	611.9812
	langshanica-xinningensis	REV+G_HKY85+G	383.31	811.7962	REV+G_RNA7E.equal	319.35	688.0881
	tenuituba-vestita	HKY85+G_HKY85+G	379.66	792.1388	HKY85+G_RNA7G.equal	305.95	642.6116
	alutacea-suichuanensis	HKY85+G_HKY85+G	338.78	710.4102	HKY85+G_RNA7G.equal	264.93	560.5976
**Nematanthus**	bradei-serpens	HKY85+G_HKY85+G	298.09	629.0751	HKY85+G_RNA7G.equal	232.85	496.4801
**Markea**	huilensis-lopezii	HKY85+G_HKY85+G	307.03	646.8970	HKY85+G_RNA7G.equal	250.69	532.1098
**Plukenetia**	huayllabambana-volubilis	HKY85+G_REV+G	319.32	683.8548	HKY85+G_RNA7G.equal	271.64	578.0050
**Maclura**	amboinensis-fruticosa	HKY85+G_HKY85+G	349.64	740.1135	HKY85+G_RNA7G.equal	289.58	617.9226
	brasiliensis-pomifera	HKY85+G_HKY85+G	365.89	776.6602	HKY85+G_RNA7G.equal	300.06	642.9340
**Delonix**	floribunda-pumila	HKY85+G_HKY85+G	251.73	548.5493	HKY85+G_RNA7G.equal	192.14	427.2713
**Caragana**	bongardiana-tragacanthoides	HKY85+G_HKY85+G	304.38	645.7245	HKY85+G_RNA7G.equal	233.51	501.8762

	opulens-licentiana	HKY85+G_HKY85+G	303.60	644.1634	HKY85+G_RNA7G.equal	230.10	495.0604
	spinosa-erinacea	HKY85+G_HKY85+G	314.75	662.4173	HKY85+G_RNA7G.equal	247.73	526.2800
**Moricandia**	arvensis-spinosa	HKY85+G_REV+G	257.83	597.3896	HKY85+G_RNA7G+G.equal	205.86	485.1275
**Meconopsis**	betonicifolia-grandis	HKY85+G_HKY85+G	374.35	785.4735	HKY85+G_RNA7G.equal	302.08	638.8471
	concinna-delavayi	HKY85+G_HKY85+G	409.79	856.3346	HKY85+G_RNA7G.equal	345.39	725.4521
	georgei_pseudovenusta	HKY85+G_HKY85+G	371.05	782.8820	HKY85+G_RNA7G.equal	307.62	653.9512
	lancifolia-impedita	HKY85+G_HKY85+G	412.34	865.4294	HKY85+G_RNA7G.equal	355.88	750.4401
	primulina-wumungensis	HKY85+G_HKY85+G	407.91	848.5417	HKY85+G_RNA7G.equal	348.15	726.9427
	quintuplinervia-punicea	HKY85+G_HKY85+G	371.64	784.0609	HKY85+G_RNA7G.equal	301.41	641.5291
	torquata-discigera	HKY85+G_HKY85+G	478.97	994.6600	HKY85+G_RNA7G.equal	424.76	884.1537
**Dichocarpum**	basilare-trifoliolatum	HKY85+G_HKY85+G	275.04	591.1166	HKY85+G_RNA7G.equal	210.95	460.8275
**Average**			343.12	733.8676		278.94	604.0172

[a] SSP is the abbreviation of sister-species pair; In order to comparable with other models, the 7-state models require a likelihood-adjustment value to account for the mapping from 1 mismatch state to 10, and each of the frequencies needs to be defined. This problem can be addressed by using empirical frequencies or by assuming that all non-canonical di-nucleotides are equally likely, which indicated as “AICc” scores according to Allen & Whelan (2014) [15]. The corresponding results are marked as “equal” after their model names.

2. Supplementary Figure

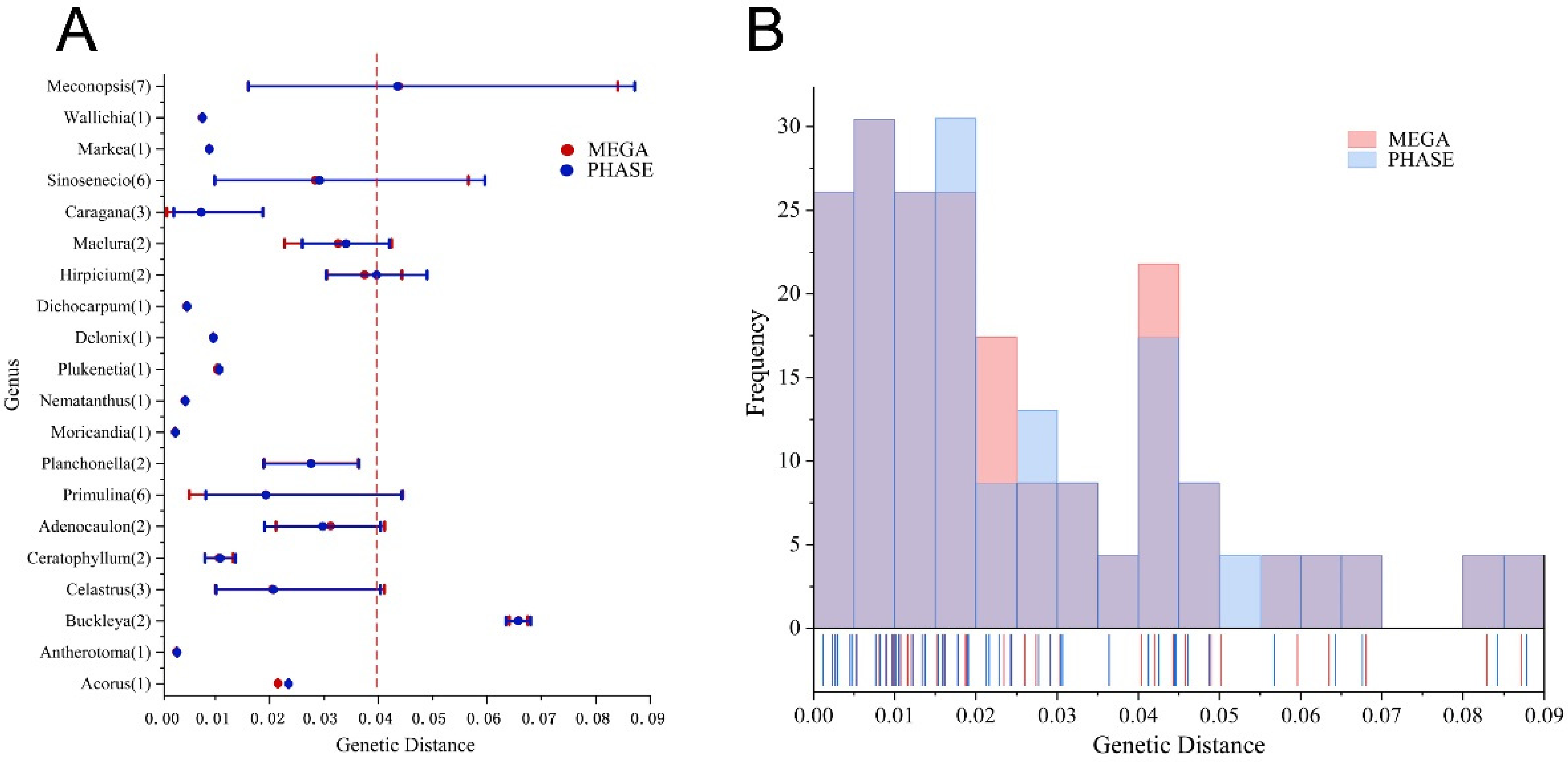

Figure S1. ITS2 genetic distances of sister-species pairs vary among different lineages. (A) Distribution of ITS2 genetic distances in 20 angiosperm lineages. Average genetic distances are shown as dots; bars represent the range of values obtained within sister species pairs, and sample sizes are shown in parentheses; (B) Histogram of ITS2 genetic distances showing the similar distribution interval between MEGA and PHASE. Genetic distances represented by different colors were generated separately by MEGA and PHASE program under the DNA substitution models.

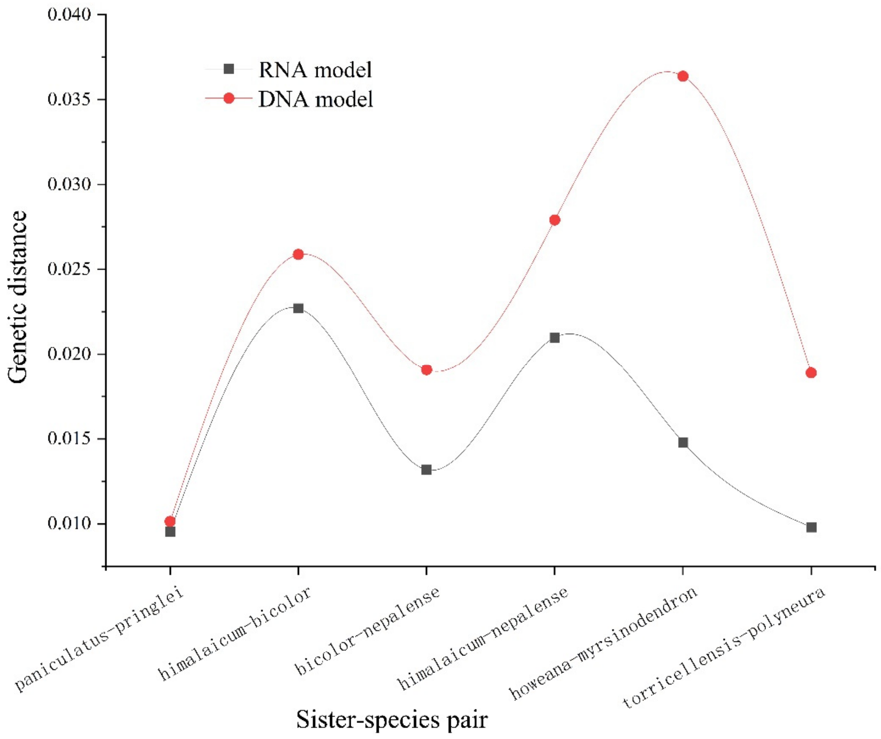

Figure S2. Comparisons of ITS2 genetic distances between RNA and DNA substitution models when the effect of compensatory base change in stem regions is too large to be neglected.
